# Trichlorido{2-dimeth­oxy­methyl-4-methyl-6-[(quinolin-8-yl)imino­meth­yl]phenolato-κ^3^ *N*,*N*′,*O* ^1^}tin(IV)

**DOI:** 10.1107/S1600536812002528

**Published:** 2012-01-31

**Authors:** Keisuke Kawamoto, Takashi Shibahara

**Affiliations:** a Department of Chemistry, Okayama University of Science, Ridai-cho, Okayama 700-0005, Japan

## Abstract

In the title compound, [Sn(C_20_H_19_N_2_O_3_)Cl_3_], the Sn^IV^ ion is surrounded by a tridentate monoanionic Schiff base and by three meridional chloride ions in a six-coordinated distorted octa­hedral geometry. The Sn—Cl bond [2.366 (2) Å] *trans* to nitro­gen is shorter than the others [2.438 (2) and 2.414 (2) Å]. The N—Sn—N angle [76.19 (11)°] is smaller than the O—Sn—N angle [87.89 (10)°] in the Schiff base ligand. No classical inter­molecular hydrogen-bonding inter­actions are observed. The crystal packing exhibits π–π stacking inter­actions, with a distance of 3.595 (2) Å between the centroids of the phenolate ring and the benzene ring of the quinoline group of inversion-related mol­ecules.

## Related literature

For a related structure, see: Takano & Shibahara (2008 ▶).
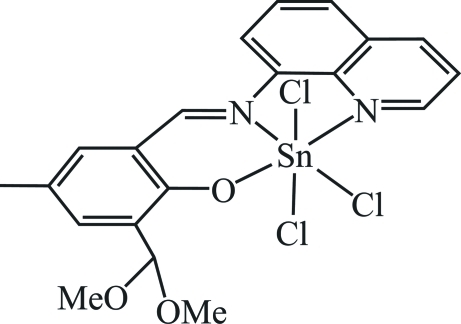


## Experimental

### 

#### Crystal data

[Sn(C_20_H_19_N_2_O_3_)Cl_3_]
*M* *_r_* = 560.43
Triclinic, 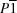

*a* = 7.213 (5) Å
*b* = 11.786 (8) Å
*c* = 13.197 (8) Å
α = 72.300 (13)°
β = 79.10 (3)°
γ = 89.89 (3)°
*V* = 1047.5 (13) Å^3^
*Z* = 2
Mo *K*α radiation
μ = 1.63 mm^−1^
*T* = 93 K
0.23 × 0.22 × 0.11 mm

#### Data collection

Rigaku Mercury70 diffractometer
Absorption correction: multi-scan (*REQAB*; Rigaku, 1998 ▶) *T*_min_ = 0.779, *T*_max_ = 0.836
15965 measured reflections
5989 independent reflections
5512 reflections with *F* ^2^ > 2σ(*F* ^2^)
*R*_int_ = 0.020

#### Refinement

*R*[*F* ^2^ > 2σ(*F* ^2^)] = 0.042
*wR*(*F* ^2^) = 0.101
*S* = 1.04
5989 reflections
292 parameters
H-atom parameters constrained
Δρ_max_ = 1.51 e Å^−3^
Δρ_min_ = −1.24 e Å^−3^

### 

Data collection: *CrystalClear* (Rigaku, 2007) ▶; cell refinement: *CrystalClear*; data reduction: *CrystalClear*; program(s) used to solve structure: *SIR2004* (Burla *et al.*, 2005 ▶); program(s) used to refine structure: *SHELXL97* (Sheldrick, 2008 ▶); molecular graphics: *CrystalStructure* (Rigaku, 2010 ▶); software used to prepare material for publication: *CrystalStructure* .

## Supplementary Material

Crystal structure: contains datablock(s) global, I. DOI: 10.1107/S1600536812002528/pk2385sup1.cif

Structure factors: contains datablock(s) I. DOI: 10.1107/S1600536812002528/pk2385Isup2.hkl

Additional supplementary materials:  crystallographic information; 3D view; checkCIF report

## Figures and Tables

**Fig. 1.**
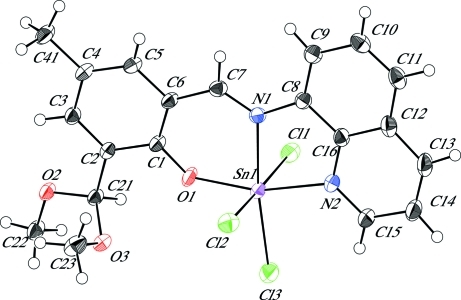
The molecular structure of the title molecule. Displacement ellipsoids are drawn at the 50% probability level.

**Fig. 2.**
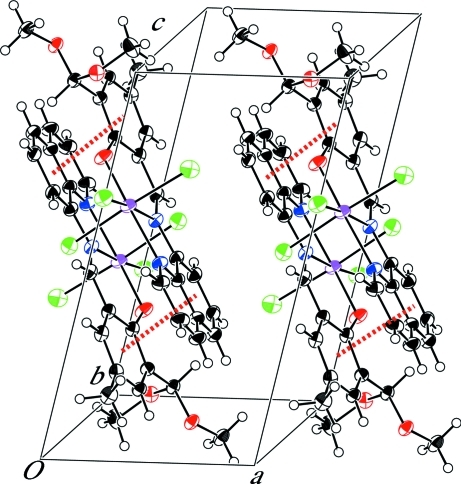
A view of the crystal packing of the title molecule showing the intermolecular π-π stacking interactions.

## supplementary crystallographic information

### Comment

The title compound is a tin(IV) complex with a Schiff base ligand, which was obtained by the condensation reaction of 8-aminoquinoline with 2-hydroxy-5-methylisophthalaldehyde. The synthetic method of the complex was reported, and the ^1^H NMR spectrum in DMSO-*d*_6_ revealed that the formyl group of the aldehyde changed to the acetal group on coordination to tin(IV) (Takano & Shibahara, 2008).

In this paper, we report the X-ray analysis of the title compound using a different synthetic method from the previous one: 1) acetylacetone tin(IV) dichloride salt, Sn^IV^Cl_2_(C_5_H_7_O_2_)_2_, was used in place of SnCl_4_.5H_2_O. 2) the new method does not require heating. 3) tetrabutyl ammonium chloride was added to supply chloride ion. The new method gives orange block crystals of the title compound by slow ligand exchange reaction from acetylacetonate to Schiff base, while the previous method gives yellow powder by heating at 60°C for 2~3 h. The ^1^H NMR spectrum of the crystals is identical to that of the yellow powder.

In the X-ray structure, the tin(IV) complex has a six-coordinated distorted octahedral geometry coordinated by a tridentate monoanionic Schiff base and by three meridional chloride ions (Fig. 1). The difference in *trans* influence between imino N and Cl induces different Sn1—Cl distances [Sn1—Cl1, 2.438 (2) Å; Sn1—Cl2, 2.414 (2) Å; Sn1—Cl3, 2.366 (2) Å]. The N1—Sn1—N2 angle [76.20 (11)°] is smaller than the O1—Sn—N1 angle [87.89 (10)°] in the Schiff base ligand. Although no intermolecular interaction *via* hydrogen bonding was observed in the crystal packing, the crystal packing exhibits π-π stacking interactions with a distance of 3.595 (2) Å between the centroids of aromatic rings (Fig. 2).

### Experimental

To a solution of acetylacetone tin(IV) dichloride salt, Sn^IV^Cl_2_(C_5_H_7_O_2_)_2_ (50.24 mg, 0.13 mmol) in methanol (60 ml) was added tetrabutyl ammonium chloride (36.72 mg, 0.13 mmol). To the methanol solution was added 8-aminoquinoline (19.09 mg, 0.13 mmol) and 2-hydroxy-5-methylisophthalaldehyde (21.58 mg, 0.13 mmol). Orange block single crystals of title compound suitable for X-ray analysis were obtained after leaving the solution to stand for three weeks at room temperature in the dark.

### Refinement

H atoms were positioned geometrically and refined using a riding model with C—H 0.98–1.06 Å and with *U*_iso_(H) = 1.2 (1.5 for methyl groups) times *U*_eq_ (C).

### Crystal data

**Table:**

[Sn(C_20_H_19_N_2_O_3_)Cl_3_]	*Z* = 2
*M**_r_* = 560.43	*F*(000) = 556.00
Triclinic, *P*1	*D*_x_ = 1.777 Mg m^−^^3^
Hall symbol: -P 1	Mo *K*α radiation, λ = 0.71070 Å
*a* = 7.213 (5) Å	Cell parameters from 3411 reflections
*b* = 11.786 (8) Å	θ = 3.0–30.0°
*c* = 13.197 (8) Å	µ = 1.63 mm^−^^1^
α = 72.300 (13)°	*T* = 93 K
β = 79.10 (3)°	Block, orange
γ = 89.89 (3)°	0.23 × 0.22 × 0.11 mm
*V* = 1047.5 (13) Å^3^	

### Data collection

**Table:**

Rigaku Mercury70 diffractometer	5512 reflections with *F*^2^ > 2σ(*F*^2^)
Detector resolution: 7.314 pixels mm^-1^	*R*_int_ = 0.020
ω scans	θ_max_ = 30.1°
Absorption correction: multi-scan (*REQAB*; Rigaku, 1998)	*h* = −10→10
*T*_min_ = 0.779, *T*_max_ = 0.836	*k* = −16→16
15965 measured reflections	*l* = −18→18
5989 independent reflections	

### Refinement

**Table:**

Refinement on *F*^2^	Secondary atom site location: difference Fourier map
*R*[*F*^2^ > 2σ(*F*^2^)] = 0.042	Hydrogen site location: inferred from neighbouring sites
*wR*(*F*^2^) = 0.101	H-atom parameters constrained
*S* = 1.04	*w* = 1/[σ^2^(*F*_o_^2^) + (0.0376*P*)^2^ + 3.612*P*] where *P* = (*F*_o_^2^ + 2*F*_c_^2^)/3
5989 reflections	(Δ/σ)_max_ = 0.001
292 parameters	Δρ_max_ = 1.51 e Å^−^^3^
0 restraints	Δρ_min_ = −1.24 e Å^−^^3^
Primary atom site location: structure-invariant direct methods	

### Special details

**Table:**

Geometry. All e.s.d.'s (except the e.s.d. in the dihedral angle between two l.s. planes) are estimated using the full covariance matrix. The cell e.s.d.'s are taken into account individually in the estimation of e.s.d.'s in distances, angles and torsion angles; correlations between e.s.d.'s in cell parameters are only used when they are defined by crystal symmetry. An approximate (isotropic) treatment of cell e.s.d.'s is used for estimating e.s.d.'s involving l.s. planes.
Refinement. Refinement was performed using all reflections. The weighted *R*-factor (*wR*) and goodness of fit (*S*) are based on *F*^2^. *R*-factor (gt) are based on *F*. The threshold expression of *F*^2^ > 2.0 σ(*F*^2^) is used only for calculating *R*-factor (gt).

### Fractional atomic coordinates and isotropic or equivalent isotropic displacement parameters (Å^2^)

**Table:**

	*x*	*y*	*z*	*U*_iso_*/*U*_eq_	
Sn(1)	0.07267 (3)	0.182356 (18)	0.625513 (16)	0.02282 (7)	
Cl(1)	−0.16518 (12)	0.22042 (7)	0.51208 (7)	0.03006 (17)	
Cl(2)	0.32132 (14)	0.15729 (8)	0.72856 (8)	0.03575 (19)	
Cl(3)	0.00261 (13)	−0.02600 (8)	0.67936 (7)	0.03285 (18)	
O(1)	−0.1098 (4)	0.2240 (2)	0.74212 (19)	0.0276 (5)	
O(2)	−0.4558 (3)	0.21297 (19)	1.02744 (18)	0.0231 (5)	
O(3)	−0.2056 (4)	0.1011 (2)	0.97814 (19)	0.0246 (5)	
N(1)	0.1674 (4)	0.3725 (3)	0.5581 (2)	0.0215 (5)	
N(2)	0.2821 (4)	0.1915 (3)	0.4789 (3)	0.0240 (5)	
C(1)	−0.1026 (5)	0.3198 (3)	0.7750 (3)	0.0210 (6)	
C(2)	−0.2156 (5)	0.3123 (3)	0.8775 (3)	0.0206 (6)	
C(3)	−0.2263 (5)	0.4110 (3)	0.9135 (3)	0.0223 (6)	
C(4)	−0.1290 (5)	0.5215 (3)	0.8520 (3)	0.0234 (6)	
C(5)	−0.0184 (5)	0.5282 (3)	0.7533 (3)	0.0232 (6)	
C(6)	0.0000 (5)	0.4288 (3)	0.7132 (3)	0.0215 (6)	
C(7)	0.1235 (5)	0.4495 (3)	0.6099 (3)	0.0227 (6)	
C(8)	0.2986 (5)	0.4024 (3)	0.4576 (3)	0.0223 (6)	
C(9)	0.3711 (5)	0.5165 (3)	0.3962 (3)	0.0259 (6)	
C(10)	0.4961 (5)	0.5361 (4)	0.2956 (3)	0.0278 (7)	
C(11)	0.5496 (5)	0.4427 (4)	0.2568 (3)	0.0290 (7)	
C(12)	0.4818 (5)	0.3242 (4)	0.3181 (3)	0.0264 (7)	
C(13)	0.5331 (5)	0.2226 (4)	0.2848 (3)	0.0318 (8)	
C(14)	0.4610 (6)	0.1114 (4)	0.3480 (3)	0.0322 (8)	
C(15)	0.3317 (5)	0.0988 (3)	0.4450 (3)	0.0284 (7)	
C(16)	0.3541 (5)	0.3044 (3)	0.4182 (3)	0.0229 (6)	
C(21)	−0.3243 (5)	0.1943 (3)	0.9417 (3)	0.0209 (6)	
C(22)	−0.5839 (5)	0.1104 (3)	1.0820 (3)	0.0277 (7)	
C(23)	−0.0791 (6)	0.1254 (4)	1.0415 (4)	0.0346 (8)	
C(41)	−0.1489 (6)	0.6271 (3)	0.8946 (3)	0.0311 (7)	
H(1)	−0.3956	0.1656	0.8899	0.0251*	
H(3)	−0.3008	0.4055	0.9811	0.0267*	
H(5)	0.0457	0.5973	0.7146	0.0278*	
H(7)	0.1751	0.5318	0.5771	0.0273*	
H(9)	0.3360	0.5838	0.4169	0.0310*	
H(10)	0.5339	0.6126	0.2570	0.0333*	
H(11)	0.6299	0.4556	0.1892	0.0348*	
H(13)	0.6057	0.2385	0.2185	0.0382*	
H(14)	0.4973	0.0435	0.3280	0.0387*	
H(15)	0.2692	0.0223	0.4854	0.0340*	
H(22A)	−0.5124	0.0414	1.1115	0.0415*	
H(22B)	−0.6531	0.0938	1.0304	0.0415*	
H(22C)	−0.6736	0.1259	1.1413	0.0415*	
H(23A)	−0.1522	0.1403	1.1063	0.0520*	
H(23B)	0.0041	0.1959	0.9983	0.0520*	
H(23C)	−0.0026	0.0567	1.0634	0.0520*	
H(41A)	−0.2017	0.6923	0.8442	0.0467*	
H(41B)	−0.0244	0.6538	0.9012	0.0467*	
H(41C)	−0.2335	0.6039	0.9659	0.0467*	

### Atomic displacement parameters (Å^2^)

**Table:**

	*U*^11^	*U*^22^	*U*^33^	*U*^12^	*U*^13^	*U*^23^
Sn(1)	0.02655 (12)	0.01881 (11)	0.02224 (11)	0.00370 (8)	0.00058 (8)	−0.00837 (8)
Cl(1)	0.0309 (4)	0.0267 (4)	0.0341 (4)	0.0029 (3)	−0.0042 (3)	−0.0130 (3)
Cl(2)	0.0421 (5)	0.0302 (4)	0.0358 (5)	0.0084 (4)	−0.0096 (4)	−0.0102 (4)
Cl(3)	0.0382 (5)	0.0279 (4)	0.0325 (4)	0.0000 (4)	−0.0018 (4)	−0.0125 (3)
O(1)	0.0318 (12)	0.0210 (11)	0.0291 (12)	−0.0022 (9)	0.0083 (10)	−0.0150 (9)
O(2)	0.0215 (11)	0.0200 (10)	0.0275 (11)	−0.0003 (8)	0.0030 (9)	−0.0116 (9)
O(3)	0.0255 (11)	0.0207 (10)	0.0301 (12)	0.0047 (9)	−0.0053 (9)	−0.0116 (9)
N(1)	0.0207 (12)	0.0181 (11)	0.0227 (12)	0.0018 (9)	0.0004 (10)	−0.0046 (9)
N(2)	0.0248 (13)	0.0240 (13)	0.0233 (12)	0.0056 (10)	−0.0009 (10)	−0.0098 (10)
C(1)	0.0198 (13)	0.0189 (13)	0.0258 (14)	0.0030 (11)	−0.0024 (11)	−0.0104 (11)
C(2)	0.0210 (14)	0.0201 (13)	0.0220 (13)	0.0040 (11)	−0.0030 (11)	−0.0091 (11)
C(3)	0.0210 (14)	0.0227 (14)	0.0237 (14)	0.0036 (11)	−0.0019 (11)	−0.0095 (11)
C(4)	0.0222 (14)	0.0196 (13)	0.0320 (16)	0.0049 (11)	−0.0053 (12)	−0.0133 (12)
C(5)	0.0217 (14)	0.0176 (13)	0.0306 (15)	0.0023 (11)	−0.0031 (12)	−0.0093 (12)
C(6)	0.0199 (14)	0.0184 (13)	0.0270 (14)	0.0046 (11)	−0.0022 (11)	−0.0095 (11)
C(7)	0.0199 (14)	0.0202 (14)	0.0264 (14)	0.0037 (11)	−0.0030 (11)	−0.0056 (11)
C(8)	0.0200 (14)	0.0228 (14)	0.0224 (14)	0.0051 (11)	−0.0017 (11)	−0.0060 (11)
C(9)	0.0220 (15)	0.0271 (16)	0.0264 (15)	0.0040 (12)	−0.0025 (12)	−0.0067 (12)
C(10)	0.0209 (15)	0.0316 (17)	0.0257 (15)	−0.0004 (13)	−0.0032 (12)	−0.0022 (13)
C(11)	0.0212 (15)	0.0399 (19)	0.0225 (15)	0.0030 (13)	−0.0015 (12)	−0.0065 (13)
C(12)	0.0220 (15)	0.0369 (17)	0.0211 (14)	0.0052 (13)	−0.0037 (11)	−0.0104 (13)
C(13)	0.0283 (17)	0.044 (2)	0.0242 (15)	0.0100 (15)	−0.0007 (13)	−0.0153 (15)
C(14)	0.0365 (19)	0.0353 (18)	0.0289 (17)	0.0123 (15)	−0.0043 (14)	−0.0172 (14)
C(15)	0.0320 (17)	0.0274 (16)	0.0269 (15)	0.0093 (13)	−0.0035 (13)	−0.0117 (13)
C(16)	0.0195 (14)	0.0278 (15)	0.0211 (13)	0.0076 (12)	−0.0030 (11)	−0.0076 (12)
C(21)	0.0188 (13)	0.0199 (13)	0.0245 (14)	0.0041 (11)	−0.0013 (11)	−0.0093 (11)
C(22)	0.0238 (15)	0.0247 (15)	0.0335 (17)	−0.0019 (12)	0.0049 (13)	−0.0137 (13)
C(23)	0.0349 (19)	0.0319 (18)	0.047 (2)	0.0103 (15)	−0.0187 (16)	−0.0195 (16)
C(41)	0.0338 (18)	0.0229 (15)	0.0400 (19)	0.0008 (13)	−0.0019 (15)	−0.0178 (14)

### Geometric parameters (Å, °)

**Table:**

Sn1—Cl1	2.4382 (16)	C9—C10	1.411 (5)
Sn1—Cl2	2.4143 (17)	C10—C11	1.374 (6)
Sn1—Cl3	2.3660 (19)	C11—C12	1.417 (5)
Sn1—O1	2.008 (3)	C12—C13	1.422 (6)
Sn1—N1	2.201 (3)	C12—C16	1.416 (5)
Sn1—N2	2.192 (3)	C13—C14	1.363 (5)
O1—C1	1.332 (5)	C14—C15	1.402 (5)
O2—C21	1.404 (4)	C3—H3	0.934
O2—C22	1.434 (4)	C5—H5	0.893
O3—C21	1.415 (4)	C7—H7	0.976
O3—C23	1.432 (6)	C9—H9	0.934
N1—C7	1.297 (5)	C10—H10	0.903
N1—C8	1.421 (4)	C11—H11	0.937
N2—C15	1.326 (6)	C13—H13	0.895
N2—C16	1.370 (4)	C14—H14	0.938
C1—C2	1.418 (5)	C15—H15	0.958
C1—C6	1.415 (4)	C21—H1	1.059
C2—C3	1.381 (5)	C22—H22A	0.980
C2—C21	1.517 (4)	C22—H22B	0.980
C3—C4	1.412 (4)	C22—H22C	0.980
C4—C5	1.373 (5)	C23—H23A	0.980
C4—C41	1.511 (6)	C23—H23B	0.980
C5—C6	1.421 (6)	C23—H23C	0.980
C6—C7	1.432 (5)	C41—H41A	0.980
C8—C9	1.385 (5)	C41—H41B	0.980
C8—C16	1.431 (6)	C41—H41C	0.980
			
Sn1···C6	3.443 (4)	C11···H5^vii^	3.0309
Cl3···C15	3.440 (4)	C11···H7^vii^	3.2990
O1···O3	2.954 (4)	C12···H5^vii^	3.4451
O1···C7	3.004 (4)	C12···H41A^vi^	3.2550
O1···C21	2.712 (4)	C13···H22C^viii^	3.0812
O2···C3	2.718 (4)	C13···H23A^viii^	3.3162
O2···C23	2.923 (5)	C13···H41A^vi^	3.1795
O3···C1	3.072 (4)	C14···H15^ix^	3.2562
O3···C3	3.499 (5)	C14···H22A^viii^	3.4349
O3···C22	2.830 (5)	C14···H22C^viii^	3.0215
N1···C1	3.032 (5)	C15···H14^ix^	3.4122
N2···C13	2.767 (5)	C15···H15^ix^	3.3697
C1···C4	2.847 (6)	C21···H10^vi^	3.4918
C2···C5	2.790 (5)	C21···H22A^iv^	3.3328
C2···C23	2.899 (5)	C21···H22B^iv^	3.3030
C3···C6	2.780 (5)	C22···H1^iv^	3.1603
C3···C23	3.538 (5)	C22···H13^ii^	3.1609
C7···C9	2.917 (5)	C22···H14^ii^	3.2658
C8···C11	2.820 (5)	C22···H22A^iv^	3.5069
C9···C12	2.818 (6)	C22···H22B^iv^	3.4792
C10···C16	2.797 (5)	C22···H23B^xiii^	3.4086
C12···C15	2.767 (5)	C22···H23C^xiii^	3.1561
C14···C16	2.763 (6)	C22···H41A^iii^	3.0891
C22···C23	3.575 (6)	C22···H41C^iii^	3.5326
Cl3···C15^i^	3.406 (5)	C23···H13^ii^	3.5034
O2···C13^ii^	3.418 (6)	C23···H22B^x^	3.0754
O2···C41^iii^	3.576 (5)	C23···H22C^x^	3.4270
O3···C22^iv^	3.289 (5)	C23···H23C^v^	2.8923
O3···C23^v^	3.407 (5)	C23···H41B^xii^	3.0520
N1···C7^vi^	3.554 (5)	C41···H22C^iii^	3.1160
C1···C9^vi^	3.402 (5)	C41···H23A^xii^	3.4958
C1···C10^vi^	3.439 (5)	C41···H23B^xii^	3.1304
C2···C10^vi^	3.428 (5)	H1···Cl2^xiii^	3.2377
C3···C10^vi^	3.573 (6)	H1···C22^iv^	3.1603
C4···C11^vi^	3.566 (6)	H1···H10^vi^	3.0369
C5···C11^vii^	3.372 (6)	H1···H22A^iv^	2.5356
C6···C8^vi^	3.574 (5)	H1···H22B^iv^	2.9587
C6···C9^vi^	3.249 (6)	H3···H11^ii^	2.9332
C7···N1^vi^	3.554 (5)	H3···H13^ii^	3.0994
C7···C7^vi^	3.585 (6)	H3···H41B^xii^	3.0192
C7···C10^vii^	3.246 (6)	H3···H41C^iii^	3.2938
C8···C6^vi^	3.574 (5)	H5···Cl1^vi^	3.0708
C9···C1^vi^	3.402 (5)	H5···C11^vii^	3.0309
C9···C6^vi^	3.249 (6)	H5···C12^vii^	3.4451
C9···C9^vii^	3.519 (6)	H5···H11^vii^	2.8481
C10···C1^vi^	3.439 (5)	H5···H13^vii^	3.5693
C10···C2^vi^	3.428 (5)	H7···Cl1^vi^	2.8031
C10···C3^vi^	3.573 (6)	H7···N1^vi^	3.3148
C10···C7^vii^	3.246 (6)	H7···C7^vi^	3.5286
C11···C4^vi^	3.566 (6)	H7···C8^vi^	3.5846
C11···C5^vii^	3.372 (6)	H7···C9^vii^	3.3861
C13···O2^viii^	3.418 (6)	H7···C10^vii^	3.1259
C13···C22^viii^	3.555 (7)	H7···C11^vii^	3.2990
C14···C22^viii^	3.589 (6)	H7···H10^vii^	3.3998
C15···Cl3^i^	3.406 (5)	H9···Cl1^vi^	2.9307
C15···C15^ix^	3.561 (6)	H9···O1^vi^	3.2541
C22···O3^iv^	3.289 (5)	H9···C1^vi^	3.2249
C22···C13^ii^	3.555 (7)	H9···C6^vi^	3.2517
C22···C14^ii^	3.589 (6)	H9···C7^vi^	3.4348
C23···O3^v^	3.407 (5)	H9···C8^vii^	3.3950
C23···C23^v^	3.567 (7)	H9···C9^vii^	3.4819
C41···O2^iii^	3.576 (5)	H10···Cl2^vii^	2.9803
Sn1···H15	3.1788	H10···N1^vii^	3.5888
Cl3···H15	2.7979	H10···C1^vi^	3.2819
O1···H1	2.4890	H10···C2^vi^	3.1272
O1···H23B	3.5467	H10···C3^vi^	3.5113
O2···H3	2.3835	H10···C6^vii^	3.4747
O2···H23A	2.6315	H10···C7^vii^	3.2530
O2···H23B	3.2795	H10···C21^vi^	3.4918
O3···H22A	2.5036	H10···H1^vi^	3.0369
O3···H22B	3.1662	H10···H7^vii^	3.3998
N1···H9	2.7348	H11···C4^vii^	3.5357
N2···H14	3.2179	H11···C5^vii^	3.0592
C1···H1	2.6825	H11···H3^viii^	2.9332
C1···H3	3.2639	H11···H5^vii^	2.8481
C1···H5	3.2529	H11···H41B^vii^	3.2889
C1···H7	3.3394	H11···H41C^viii^	2.9130
C1···H23B	3.1049	H13···O2^viii^	2.7444
C2···H23A	3.1988	H13···C22^viii^	3.1609
C2···H23B	2.5593	H13···C23^viii^	3.5034
C3···H1	3.2644	H13···H3^viii^	3.0994
C3···H5	3.1823	H13···H5^vii^	3.5693
C3···H23A	3.5367	H13···H22A^viii^	3.2521
C3···H23B	3.0662	H13···H22C^viii^	2.9099
C3···H41A	3.1554	H13···H23A^viii^	2.5762
C3···H41B	3.1593	H13···H41A^vi^	3.2147
C3···H41C	2.5674	H13···H41B^vii^	3.1965
C5···H3	3.2142	H14···Cl2^ix^	2.9259
C5···H7	2.4637	H14···Cl3^ix^	3.5973
C5···H41A	2.7804	H14···C15^ix^	3.4122
C5···H41B	2.7800	H14···C22^viii^	3.2658
C5···H41C	3.3087	H14···H15^ix^	3.1470
C7···H5	2.5358	H14···H22A^viii^	2.8791
C7···H9	2.7237	H14···H22C^viii^	2.8675
C8···H7	2.5553	H14···H23A^viii^	3.3818
C8···H10	3.2184	H15···Cl1^i^	2.9475
C9···H7	2.5905	H15···Cl3^i^	3.1825
C9···H11	3.2749	H15···C14^ix^	3.2562
C11···H9	3.2400	H15···C15^ix^	3.3697
C11···H13	2.6214	H15···H14^ix^	3.1470
C12···H10	3.2507	H15···H15^ix^	3.4444
C12···H14	3.2738	H22A···Cl2^v^	3.1499
C13···H11	2.6807	H22A···O3^iv^	3.2379
C13···H15	3.2430	H22A···C14^ii^	3.4349
C15···H13	3.2149	H22A···C21^iv^	3.3328
C16···H9	3.2896	H22A···C22^iv^	3.5069
C16···H11	3.2760	H22A···H1^iv^	2.5356
C16···H13	3.2054	H22A···H13^ii^	3.2521
C16···H15	3.1978	H22A···H14^ii^	2.8791
C21···H3	2.7048	H22A···H22A^iv^	3.3440
C21···H22A	2.5520	H22A···H22B^iv^	2.9030
C21···H22B	2.5528	H22B···O3^iv^	2.5561
C21···H22C	3.1846	H22B···C21^iv^	3.3030
C21···H23A	2.6156	H22B···C22^iv^	3.4792
C21···H23B	2.6165	H22B···C23^xiii^	3.0754
C21···H23C	3.2261	H22B···H1^iv^	2.9587
C22···H1	2.5279	H22B···H22A^iv^	2.9030
C22···H23A	3.2207	H22B···H22B^iv^	3.2856
C23···H1	3.2540	H22B···H23B^xiii^	2.7957
C23···H3	3.5994	H22B···H23C^xiii^	2.4914
C23···H22A	3.1568	H22B···H41A^iii^	3.4733
C41···H3	2.6551	H22C···Cl3^iv^	2.9685
C41···H5	2.6417	H22C···C13^ii^	3.0812
H1···H3	3.5070	H22C···C14^ii^	3.0215
H1···H22A	2.8057	H22C···C23^xiii^	3.4270
H1···H22B	2.3138	H22C···C41^iii^	3.1160
H1···H22C	3.4335	H22C···H13^ii^	2.9099
H1···H23A	3.5643	H22C···H14^ii^	2.8675
H1···H23B	3.5155	H22C···H23A^xiii^	3.5648
H3···H23A	3.3418	H22C···H23B^xiii^	3.2169
H3···H23B	3.2895	H22C···H23C^xiii^	2.9647
H3···H41A	3.3170	H22C···H41A^iii^	2.3690
H3···H41B	3.3166	H22C···H41B^iii^	3.3694
H3···H41C	2.3288	H22C···H41C^iii^	3.1940
H5···H7	2.2295	H23A···Cl2^v^	3.5919
H5···H41A	2.7139	H23A···Cl3^v^	3.1320
H5···H41B	2.6993	H23A···C13^ii^	3.3162
H5···H41C	3.5623	H23A···C41^xii^	3.4958
H7···H9	2.1154	H23A···H13^ii^	2.5762
H9···H10	2.2441	H23A···H14^ii^	3.3818
H10···H11	2.3396	H23A···H22C^x^	3.5648
H11···H13	2.4698	H23A···H41A^xii^	3.5024
H13···H14	2.3537	H23A···H41B^xii^	2.7087
H14···H15	2.3407	H23B···C22^x^	3.4086
H22A···H23A	2.8302	H23B···C41^xii^	3.1304
Cl1···H5^vi^	3.0708	H23B···H22B^x^	2.7957
Cl1···H7^vi^	2.8031	H23B···H22C^x^	3.2169
Cl1···H9^vi^	2.9307	H23B···H23C^v^	3.3169
Cl1···H15^i^	2.9475	H23B···H41A^xii^	3.2986
Cl2···H1^x^	3.2377	H23B···H41B^xii^	2.5342
Cl2···H10^vii^	2.9803	H23B···H41C^xii^	3.0848
Cl2···H14^ix^	2.9259	H23C···Cl2^v^	3.5506
Cl2···H22A^v^	3.1499	H23C···Cl3^v^	3.3025
Cl2···H23A^v^	3.5919	H23C···O3^v^	2.5118
Cl2···H23C^v^	3.5506	H23C···C22^x^	3.1561
Cl3···H14^ix^	3.5973	H23C···C23^v^	2.8923
Cl3···H15^i^	3.1825	H23C···H22B^x^	2.4914
Cl3···H22C^iv^	2.9685	H23C···H22C^x^	2.9647
Cl3···H23A^v^	3.1320	H23C···H23B^v^	3.3169
Cl3···H23C^v^	3.3025	H23C···H23C^v^	2.4340
Cl3···H41A^xi^	3.5133	H23C···H41B^xii^	3.5922
O1···H9^vi^	3.2541	H41A···Cl3^xiv^	3.5133
O2···H13^ii^	2.7444	H41A···O2^iii^	3.1263
O2···H41A^iii^	3.1263	H41A···C12^vi^	3.2550
O2···H41C^iii^	3.1164	H41A···C13^vi^	3.1795
O3···H22A^iv^	3.2379	H41A···C22^iii^	3.0891
O3···H22B^iv^	2.5561	H41A···H13^vi^	3.2147
O3···H23C^v^	2.5118	H41A···H22B^iii^	3.4733
N1···H7^vi^	3.3148	H41A···H22C^iii^	2.3690
N1···H10^vii^	3.5888	H41A···H23A^xii^	3.5024
C1···H9^vi^	3.2249	H41A···H23B^xii^	3.2986
C1···H10^vi^	3.2819	H41B···C3^xii^	3.2191
C2···H10^vi^	3.1272	H41B···C23^xii^	3.0520
C3···H10^vi^	3.5113	H41B···H3^xii^	3.0192
C3···H41B^xii^	3.2191	H41B···H11^vii^	3.2889
C4···H11^vii^	3.5357	H41B···H13^vii^	3.1965
C5···H11^vii^	3.0592	H41B···H22C^iii^	3.3694
C6···H9^vi^	3.2517	H41B···H23A^xii^	2.7087
C6···H10^vii^	3.4747	H41B···H23B^xii^	2.5342
C7···H7^vi^	3.5286	H41B···H23C^xii^	3.5922
C7···H9^vi^	3.4348	H41C···O2^iii^	3.1164
C7···H10^vii^	3.2530	H41C···C22^iii^	3.5326
C8···H7^vi^	3.5846	H41C···H3^iii^	3.2938
C8···H9^vii^	3.3950	H41C···H11^ii^	2.9130
C9···H7^vii^	3.3861	H41C···H22C^iii^	3.1940
C9···H9^vii^	3.4819	H41C···H23B^xii^	3.0848
C10···H7^vii^	3.1259		
			
Cl1—Sn1—Cl2	175.84 (3)	C12—C13—C14	120.5 (4)
Cl1—Sn1—Cl3	91.63 (4)	C13—C14—C15	119.1 (4)
Cl1—Sn1—O1	89.63 (10)	N2—C15—C14	121.9 (3)
Cl1—Sn1—N1	88.80 (9)	N2—C16—C8	119.1 (3)
Cl1—Sn1—N2	87.42 (10)	N2—C16—C12	120.7 (4)
Cl2—Sn1—Cl3	91.91 (5)	C8—C16—C12	120.3 (3)
Cl2—Sn1—O1	91.92 (10)	O2—C21—O3	112.4 (3)
Cl2—Sn1—N1	87.40 (9)	O2—C21—C2	108.0 (3)
Cl2—Sn1—N2	90.04 (10)	O3—C21—C2	113.1 (3)
Cl3—Sn1—O1	99.80 (8)	C2—C3—H3	119.462
Cl3—Sn1—N1	172.30 (8)	C4—C3—H3	117.886
Cl3—Sn1—N2	96.15 (9)	C4—C5—H5	117.907
O1—Sn1—N1	87.89 (11)	C6—C5—H5	119.889
O1—Sn1—N2	163.86 (10)	N1—C7—H7	119.449
N1—Sn1—N2	76.19 (11)	C6—C7—H7	113.472
Sn1—O1—C1	128.15 (19)	C8—C9—H9	122.470
C21—O2—C22	111.3 (3)	C10—C9—H9	116.978
C21—O3—C23	114.7 (3)	C9—C10—H10	116.927
Sn1—N1—C7	123.33 (19)	C11—C10—H10	122.110
Sn1—N1—C8	114.4 (3)	C10—C11—H11	121.166
C7—N1—C8	122.0 (3)	C12—C11—H11	118.342
Sn1—N2—C15	124.7 (2)	C12—C13—H13	115.150
Sn1—N2—C16	114.7 (3)	C14—C13—H13	124.102
C15—N2—C16	120.5 (3)	C13—C14—H14	121.206
O1—C1—C2	116.5 (3)	C15—C14—H14	119.693
O1—C1—C6	124.9 (3)	N2—C15—H15	118.475
C2—C1—C6	118.6 (3)	C14—C15—H15	119.436
C1—C2—C3	119.8 (3)	O2—C21—H1	110.026
C1—C2—C21	117.3 (3)	O3—C21—H1	104.487
C3—C2—C21	122.9 (3)	C2—C21—H1	108.782
C2—C3—C4	122.7 (3)	O2—C22—H22A	109.469
C3—C4—C5	117.4 (4)	O2—C22—H22B	109.470
C3—C4—C41	120.3 (3)	O2—C22—H22C	109.471
C5—C4—C41	122.2 (3)	H22A—C22—H22B	109.473
C4—C5—C6	122.2 (3)	H22A—C22—H22C	109.468
C1—C6—C5	119.4 (3)	H22B—C22—H22C	109.476
C1—C6—C7	125.2 (4)	O3—C23—H23A	109.471
C5—C6—C7	115.5 (3)	O3—C23—H23B	109.472
N1—C7—C6	127.1 (3)	O3—C23—H23C	109.474
N1—C8—C9	125.4 (4)	H23A—C23—H23B	109.469
N1—C8—C16	115.5 (3)	H23A—C23—H23C	109.474
C9—C8—C16	119.1 (3)	H23B—C23—H23C	109.469
C8—C9—C10	120.5 (4)	C4—C41—H41A	109.468
C9—C10—C11	120.9 (3)	C4—C41—H41B	109.471
C10—C11—C12	120.5 (3)	C4—C41—H41C	109.471
C11—C12—C13	124.0 (3)	H41A—C41—H41B	109.469
C11—C12—C16	118.8 (4)	H41A—C41—H41C	109.478
C13—C12—C16	117.2 (3)	H41B—C41—H41C	109.469
			
Cl1—Sn1—O1—C1	−109.7 (2)	O1—C1—C2—C21	3.3 (4)
Cl1—Sn1—N1—C7	102.3 (2)	O1—C1—C6—C5	173.9 (3)
Cl1—Sn1—N1—C8	−84.36 (17)	O1—C1—C6—C7	−5.4 (5)
Cl1—Sn1—N2—C15	−90.7 (3)	C2—C1—C6—C5	−2.3 (5)
Cl1—Sn1—N2—C16	85.53 (18)	C2—C1—C6—C7	178.3 (3)
Cl2—Sn1—O1—C1	66.5 (2)	C6—C1—C2—C3	1.3 (5)
Cl2—Sn1—N1—C7	−79.4 (2)	C6—C1—C2—C21	179.8 (3)
Cl2—Sn1—N1—C8	93.95 (17)	C1—C2—C3—C4	0.3 (5)
Cl2—Sn1—N2—C15	92.6 (3)	C1—C2—C21—O2	−167.6 (3)
Cl2—Sn1—N2—C16	−91.18 (19)	C1—C2—C21—O3	67.5 (4)
Cl3—Sn1—O1—C1	158.73 (18)	C3—C2—C21—O2	10.9 (5)
Cl3—Sn1—N2—C15	0.7 (3)	C3—C2—C21—O3	−114.1 (4)
Cl3—Sn1—N2—C16	176.89 (18)	C21—C2—C3—C4	−178.1 (3)
O1—Sn1—N1—C7	12.7 (2)	C2—C3—C4—C5	−0.8 (5)
O1—Sn1—N1—C8	−174.03 (19)	C2—C3—C4—C41	178.8 (3)
N1—Sn1—O1—C1	−20.9 (2)	C3—C4—C5—C6	−0.2 (5)
N1—Sn1—N2—C15	179.9 (3)	C41—C4—C5—C6	−179.9 (3)
N1—Sn1—N2—C16	−3.87 (18)	C4—C5—C6—C1	1.8 (5)
N2—Sn1—N1—C7	−170.0 (3)	C4—C5—C6—C7	−178.8 (3)
N2—Sn1—N1—C8	3.27 (17)	C1—C6—C7—N1	−2.7 (6)
Sn1—O1—C1—C2	−162.50 (17)	C5—C6—C7—N1	177.9 (3)
Sn1—O1—C1—C6	21.2 (5)	N1—C8—C9—C10	−177.8 (3)
C22—O2—C21—O3	−63.5 (4)	N1—C8—C16—N2	−1.1 (5)
C22—O2—C21—C2	171.0 (3)	N1—C8—C16—C12	179.0 (3)
C23—O3—C21—O2	−66.9 (3)	C9—C8—C16—N2	−179.9 (3)
C23—O3—C21—C2	55.7 (4)	C9—C8—C16—C12	0.2 (5)
Sn1—N1—C7—C6	−4.5 (5)	C16—C8—C9—C10	0.8 (5)
Sn1—N1—C8—C9	176.4 (3)	C8—C9—C10—C11	−0.6 (6)
Sn1—N1—C8—C16	−2.3 (4)	C9—C10—C11—C12	−0.7 (6)
C7—N1—C8—C9	−10.2 (5)	C10—C11—C12—C13	−178.8 (3)
C7—N1—C8—C16	171.1 (3)	C10—C11—C12—C16	1.7 (6)
C8—N1—C7—C6	−177.3 (3)	C11—C12—C13—C14	−179.6 (4)
Sn1—N2—C15—C14	177.3 (2)	C11—C12—C16—N2	178.7 (3)
Sn1—N2—C16—C8	4.0 (4)	C11—C12—C16—C8	−1.4 (5)
Sn1—N2—C16—C12	−176.08 (19)	C13—C12—C16—N2	−0.9 (5)
C15—N2—C16—C8	−179.6 (3)	C13—C12—C16—C8	179.0 (3)
C15—N2—C16—C12	0.3 (5)	C16—C12—C13—C14	−0.1 (6)
C16—N2—C15—C14	1.3 (6)	C12—C13—C14—C15	1.6 (6)
O1—C1—C2—C3	−175.3 (3)	C13—C14—C15—N2	−2.2 (6)

Symmetry codes: (i) −*x*, −*y*, −*z*+1; (ii) *x*−1, *y*, *z*+1; (iii) −*x*−1, −*y*+1, −*z*+2; (iv) −*x*−1, −*y*, −*z*+2; (v) −*x*, −*y*, −*z*+2; (vi) −*x*, −*y*+1, −*z*+1; (vii) −*x*+1, −*y*+1, −*z*+1; (viii) *x*+1, *y*, *z*−1; (ix) −*x*+1, −*y*, −*z*+1; (x) *x*+1, *y*, *z*; (xi) *x*, *y*−1, *z*; (xii) −*x*, −*y*+1, −*z*+2; (xiii) *x*−1, *y*, *z*; (xiv) *x*, *y*+1, *z*.
